# Heat stress during seed filling interferes with sulfur restriction on grain composition and seed germination in oilseed rape (*Brassica napus* L.)

**DOI:** 10.3389/fpls.2015.00213

**Published:** 2015-04-10

**Authors:** Sophie Brunel-Muguet, Philippe D'Hooghe, Marie-Paule Bataillé, Colette Larré, Tae-Hwan Kim, Jacques Trouverie, Jean-Christophe Avice, Philippe Etienne, Carolyne Dürr

**Affiliations:** ^1^INRA, UMR INRA–UCBN 950 Ecophysiologie Végétale, Agronomie et Nutritions N.C.S.Caen, France; ^2^UCBN, UMR INRA–UCBN 950 Ecophysiologie Végétale, Agronomie et Nutritions N.C.S.Caen, France; ^3^INRA UR 1268 BIA, Rue de la GéraudièreNantes, France; ^4^Department of Animal Science, Institute of Agricultural Science and Technology, College of Agriculture and Life Science, Chonnam National UniversityGwangju, South Korea; ^5^INRA, UMR 1345, Institute of Research on Horticulture and Seeds, SFR QUASAVBeaucouzé, France

**Keywords:** oilseed rape, sulfur, temperature, germination, grain quality

## Abstract

In coming decades, increasing temperatures are expected to impact crop yield and seed quality. To develop low input systems, the effects of temperature and sulfur (S) nutrition in oilseed rape, a high S demanding crop, need to be jointly considered. In this study, we investigated the effects of temperatures [High Temperature (HT), 33°C/day, 19°C/night *vs*. Control Temperature (Ctrl T), 20°C/day, 15°C/day] and S supply [High S (HS), 500 μm SO^2−^_4_ *vs*. Low S (LS), 8.7 μM SO^2−^_4_] during seed filling on (i) yield components [seed number, seed dry weight (SDW) and seed yield], (ii) grain composition [nitrogen (N) and S contents] and quality [fatty acid (FA) composition and seed storage protein (SSP) accumulation] and (iii) germination characteristics (pre-harvest sprouting, germination rates and abnormal seedlings). Abscisic acid (ABA), soluble sugar contents and seed conductivity were also measured. HT and LS decreased the number of seeds per plant. SDW was less affected due to compensatory effects since the number of seeds decreased under stress conditions. While LS had negative effects on seed composition by reducing the FA contents and increasing the ratio S-poor SSPs (12S globulins)/S-rich SSPs (2S albumins) ratio, HT had positive effects by increasing S and FA contents and decreasing the C18:2/C18:3 ratio and the 12S/2S protein ratio. Seeds produced under HT showed high pre-harvest sprouting rates along with decreased ABA contents and high rates of abnormal seedlings. HT and LS restriction significantly accelerated germination times. High conductivity, which indicates poor seed storage capacity, was higher in HT seeds. Consistently, the lower ratio of (raffinose + stachyose)/sucrose in HT seeds indicated low seed storage capacity. We demonstrated the effects of HT and LS on grain and on germination characteristics. These results suggest that hormonal changes might control several seed characteristics simultaneously.

## Introduction

Attempts to reduce crop dependency on fertilizers by increasing the efficiency of nutrient use are hampered by unknown impacts of climate change, especially those of heat stress during the grain filling period of crops (Luzuriaga et al., 2006; Hedhly et al., 2009; Huang et al., 2014). Seed filling involves massive synthesis and deposition of reserves. In the case of oilseed rape, these reserves are mainly composed of oil (45–50% w/w) and proteins (20–25% w/w). Oil is the main product for food uses and the composition of fatty acids (FAs) needs to be tightly controlled to meet nutritional requirements. Moreover, 60% of seed proteins in oilseed rape are seed storage proteins (SSP) constituted by 12S globulins (cruciferins) and 2S albumins (napins), both sulfur (S) containing amino acids [cysteine (Cys) and methionine (Met)], which contribute respectively to 2.5% (cruciferins) and 10% (napins) of total seed amino acids (Schwenke et al., 1981; Monsalve et al., 1991). High levels of S-rich proteins, with high concentrations of essential amino acids Cys and Met (Kohno-Murase et al., 1995; D'Hooghe et al., 2014) are usually targeted. Like most *Brassicaceae* species, oilseed rape, the third largest oil crop worldwide (http://www.fao.org/statistics/), is a high S-demanding crop and requires up to four times more S than wheat (Postma et al., 1999; source: Aspach DGER 1992). S fertilization has been the subject of growing attention in recent decades due to increasing areas of S deficient crops observed in Europe (Schnug et al., 1993; Scherer, 2001; McGrath et al., 2002). This deficiency is the result of legislation in the 1980s aiming at decreasing the level of sulfur dioxide in industrial emissions, which drastically reduced these “free” S fertilizer depositions (Schnug and Evans, 1992). It is also the consequence of the substitution of S-containing fertilizers and S-containing fungicides with alternative compounds containing no or only small amounts of S (Zhao et al., 1999). S limitation may lead to 40% of seed yield in oilseed rape because of pod abortions (Postma et al., 1999).

Many studies have focused on climatic and environmental factors and dealt with the combination of high temperatures with CO_2_ and/or an increase in ozone (for a review on heat stress see Wahid et al., 2007; and on oilseed rape: Högy et al., 2010; Frenck et al., 2011). Few studies included fertilization constraints even though interactions may occur (Nitrogen: Franzaring et al., 2012; Andrianasolo et al., 2014). Those studies on oilseed rape which analyzed the effects of S restriction (McGrath and Zhao, 1996; Ahmad and Abdin, 2000; Dubousset et al., 2010; D'Hooghe et al., 2013, 2014; Girondé et al., 2014) or heat stress (Canvin, 1965; Baux et al., 2008, 2013) were focused on seed FA content, accumulation of SSP and on sugar and S metabolism. The few studies on the effects of heat stress reported decreases in the proportion of unsaturated FAs (Canvin, 1965). When S was restricted during the early reproductive phase, oilseed rape had lower contents of lipids, mainly C18 derivatives, and of S-rich SSP (Dubousset et al., 2010; D'Hooghe et al., 2014). Changes in the accumulation of soluble sugars involved in the acquisition of drying tolerance were also observed e.g., increases in glucose and raffinose contents but without changes in sucrose contents (in *Arabidopsis*, Nikiforova et al., 2005). Soluble sugars help maintain cell membrane integrity during the seed desiccation stage and high seed storage ability has been shown to be correlated with a high (raffinose + stachyose)/sucrose ratio in seeds (maize, Bochicchio et al., 1997; bean, Bailly et al., 2001). High seed conductivity values (estimated by electrolyte leakage when the seed is immersed in demineralized water) indicate poor cell membrane integrity. High values are also correlated with poor seed storability (ISTA, 2013) as well as with poor field emergence (Matthews and Powell, 2006; Demir et al., 2012; Mavi et al., 2014). No studies on seed lots grown under both S restriction and heat stress during pod formation have focused on other essential seed characteristics such as germination and seed storage capacity. These characteristics could be analyzed together with changes in grain composition, and also combined with measurements of plant growth regulators, especially abscisic acid (ABA) and gibberellic acid (GAs). ABA is involved in seed development programming with respect to germination suppression, accumulation of reserves and desiccation induction (Koornneef et al., 1982; White et al., 2000). ABA content in seeds increases during SSP deposition and then decreases during seed desiccation, and low ABA content is a precondition for seed germination (Crouch and Sussex, 1981; Finkelstein et al., 1985; Rajjou et al., 2011). Exogenous ABA was shown to enhance the accumulation of soluble sugars followed by the deposition of lipids (Chandrasekaran et al., 2014). GAs are known to be involved in cell expansion and to counterbalance the effects of ABA on germination by negatively regulating germination repressors (for review see Brian, 2008; Rajjou et al., 2011).

In our study, we thus investigated the effects of both high temperatures and S availability during the grain filling period (from flowering until the end of pod ripening) and their interactions, on grain yield and composition, and on seed characteristics related to germination and storage capacities. We measured (i) yield components [number of reproductive branches, number of seeds, seed dry weight (SDW), and seed yield], (ii) grain composition (C, N, S, FA, SSP contents), (iii) seed germination characteristics (pre-harvest sprouting, germination at sub- and optimum temperatures and abnormal seedlings). ABA, GA, soluble sugar contents, and conductivity were also measured as complementary seed characteristics. The impacts of both types of stress on yield components and grain quality are discussed in light of the hormonal changes that occurred during grain filling.

## Materials and methods

### Experimental treatments and growth conditions

Seeds of *Brassica napus* L. (cv. Aviso) were sown in perlite with a thermoperiod of 20°C (16 h day) and 15°C (8 h night) in October 2011, for 60 days on a 25% Hoagland solution consisting of 1.25 mM Ca(CO_3_)_2_, 4H_2_O, 1.25 mM KNO_3_, 0.5 mM MgSO_4_, 0.25 mM KH_2_PO_4_, 0.2 mM Fe-Na EDTA, 14 μM H_3_BO_3_, 5μM MnSO_4_, 3 μM ZnSO_4_, 0.7 μM (NH_4_)_6_Mo_7_O_24_, 0.7 μM CuSO_4_, 0.1 μM CoCl2. As described in Dubousset et al. (2010), seedlings were then subjected to a 10-week period of vernalization in a climatic chamber maintained at 4°C (night) and 8°C (day) with artificial light during the day (10 h day/14 h night). After this period of vernalization, each plant was transferred into a pot containing perlite and vermiculite (2:1, v/v) and subjected to a thermoperiod of 20°C (day) and 15°C (night) with natural light (greenhouse located at the University of Caen, 49°11′09 N, 0°21′32 W). Plants were automatically supplied with increasing volumes of the nutrient solution (25% Hoagland). The contrasting S supplies (non-limiting in the high S, HS treatment, and S-limiting in the low S, LS treatment) were applied at the beginning of pollination when the old petals started falling (GS70 according to the BBCH system, Lancashire et al., 1991). Ten plants per treatment were supplied with a solution containing 500 and 8.7 μM SO^2−^_4_ (2% of the non-limiting S condition) in the HS and LS treatments, respectively. The solution was ^34^S labeled (2 atom% excess) from the end of vernalization until GS70 in order to measure the contribution of S uptake from GS16 (rosette stage) to GS70 to final S contents in the seed. High temperatures were applied from the beginning of pod formation (GS81) until pod maturity (GS99). The plants subjected to high temperatures were moved to a separate greenhouse where the average day temperature was 32.9°C (±2.0) for 16 h and the average night temperature was 19.3°C (±1.6) for 8 h throughout the experiment (Morrison and Stewart, 2002). These conditions corresponded to very high temperatures compared to field conditions since oilseed rape is predominantly cultivated in temperate conditions. Temperatures were recorded hourly with temperature probes (105T Campbell, Campbell Scientific Ltd., Leicestershire, UK). Five plants were grown and harvested at maturity in the four treatments i.e., control temperature/high S (Ctrl T/HS), control temperature/low S (Ctrl T/LS), high temperature/high S (HT/HS) and high temperature/low S (HT/LS).

### Yield components

Plants were harvested at maturity, i.e., when all the pods were dry and the seeds were dark and hard (1285° Cd for Ctrl T and 2140° Cd for HT). The dry weight of all the seeds harvested per plant (seed yield), the SDW were measured as well as the number of final branches without pods or with mainly aborted pods (<2 cm length), or with mainly non-aborted pods (>2 cm length), and number of pods per length class on the main stem.

### Seed biochemical characteristics

#### C, N, and S seed contents

Seeds of each individual plant were pooled and dried for 48 h at 50°C. The dried seeds were ground and the resulting powder (3 mg) was placed in tin capsules for analysis. The total C, N, and S contents of the dried seeds were determined by a C/N/S analyzer (EA3000, Euro Vector, Milan, Italy) linked to a continuous flow isotope mass spectrometer (IRMS, Isoprime, GV Instrument, Manchester, UK). The amounts of C (Q_C_), N (Q_N_), and S (Q_S_) were calculated as follows:

$$\begin{array}{l} {\text{Q}}_{\text{C}}\;=\%\text{C}\times \text{DW}/100,\;{\text{Q}}_{\text{N}}\text{=}\%\text{N}\times \text{DW}/100\text{ and }{\text{Q}}_{\text{S}}\\ \;=\%\text{S}\times \text{DW}/100 \end{array}$$

where %C, %N, and %S were the C, N, and S contents within the seed tissue given by mass spectrometry. IRMS analysis also provided the amount of ^34^S in excess (E%) in the sample to be quantified as follows:

$$\text{E}\%=(\text{A}\%)\;-\text{ A}{\%}_{\text{stand}}$$

where A% is the tissue ^34^S abundance given by mass spectrometry and A%_stand_ is the isotope abundance in the natural standard (4.2549% for unlabeled oilseed rape). Therefore, the amount of ^34^S (Q_34S_) can be calculated as follows:

$${\text{Q}}_{34\text{S}}=\text{E}\%\times {\text{Q}}_{\text{S}}$$

Since the amount of ^34^S was taken up from GS16 to GS70 i.e., before pod formation, the Q_34S_ in the seeds at harvest originated from remobilization fluxes from the leaves, stem, inflorescences and belowground parts to seeds that occurred after GS70. Therefore, the calculation of ^34^S incorporated in seeds (Q^34^S%) indicates the incorporation rate to the seeds of the S amount taken up before pod formation.

#### Fatty acid contents

One hundred milligrams of seeds from each of the five plants in the four treatments were ground and suspended in 1.5 mL of methanol/H_2_SO_4_ solution (100:2.5; v/v) containing C17:0 as internal standard (5 μg mL^−1^) overnight at 85°C for transmethylation. After cooling, 500 μL of hexane was added and the hexane phase containing the resulting fatty acid methyl esters (FAMES) was recovered for gas chromatography analysis combined with flame ionization detection (GC-FID). The FAMES (1 μL) were injected into an Agilent 7890 gas chromatograph equipped with a Carbowax column (15 m by 0.53 mm, 1.2 m) (Alltech Associates, Deerfield,IL) and FID system. The temperature gradient was 160°C for 1 min, increased to 190°C by 20°C min^−1^, increased to 210°C by 5°C min^−1^, and then maintained at 210°C for 5 min. FAMES were identified by comparing their retention times with those of commercial standards (Sigma, St. Louis, MO): C16:0 (palmitic acid), C18:0 (stearic acid), C18:1 (oleic acid), C18:2 (linoleic acid), C18:3 (linolenic acid), C20:0 (arachidic acid), C20:1 (gadoleic acid), and C22:1 (erucic acid). FAMES were quantified using ChemStation (Agilent) to calculate the peak areas. The internal standard (C17:0) was then used to compare samples.

#### Soluble sugar contents

For each treatment, soluble sugars were extracted from three replicates of 18 mg from lyophilized and ground seeds (pooled from the five plants), with 1 mL methanol/water (80:20; v/v) and 40 μL melicitose was used as the internal sugar standard. After heating for 15 min at 76°C, the liquid was evaporated under vacuum. The pellet was then dissolved in 1 mL distilled water and centrifuged for 3 min at 14,000 *g*. Glucose, fructose, sucrose, raffinose, and stachyose contents were quantified using HPLC (DIONEX ICS-3000).

#### Seed storage protein contents

For each treatment, seeds were pooled from the five plants (2.5 g) and ground in a cryogenic apparatus (Fritsch Spartan) for 4 min. Dichloromethane (1:10; w/v) was then added to the crushed seeds and the mixture stirred for 10 min at room temperature. The suspension was then centrifuged at 15,000 *g*, the supernatant removed and the pellet dried at room temperature in a fume hood. The dried and defatted pellet was then manually crushed with a pestle and mortar and stored at −20°C. Defatted rapemeal (15 mg) was shaken in 1.5 mL of 50 mM Tris–HCl buffer, pH 8.5, containing 750 mM NaCl, 5 mM EDTA and 15 mM sodium bisulfite for 2 h. Proteins and pigments were recovered in the supernatant resulting from centrifugation of the suspension at 15,000 *g* for 10 min. The supernatant (1 mL) was further depigmented by desalting chromatography on a PD10 column (GE Healthcare) and the resulting protein-rich fraction (200 μL) was separated using a Superdex 75 column (24 mL, Pharmacia) eluted with 50 mM Tris–HCl buffer, pH 8.5, containing 750 mM NaCl and 5 mM EDTA. Elution was carried out at 1 mL/min, detection was performed at 280 nm and the eluted fractions collected. The nature and quality of each eluted peak was checked by SDS-PAGE electrophoresis according to Bérot et al. (2005). The chromatographic profiles were recorded, normalized, and the area under each peak was integrated. Cruciferins (12S globulin) and napins (2S albumin) were quantified. Extractions were performed in triplicate.

#### Abscisic acid (ABA) and gibberellic acid (GA_3_) measurements

For each replicate of each treatment, 50 mg of ground seeds were collected to quantify 2-cis, 4-trans- ABA and gibberellic acid (GA_3_) The ABA/GA ratio is an indicator of the potential for seed dormancy and hence seed storage capacity. Previously freeze dried samples were mixed with 500 μL of extraction solvent [2-propanol/H_2_O/ concentrated HCl (2:1:0.002; v/v/v)] and then analyzed by HPLC-MS as described in Pan et al. (2010).

### Seed characteristics

#### Pre-harvest sprouting, germination at sub- and optimum temperatures and abnormal seedlings

A subsample of seeds from each plant was collected just after harvest (30–130 seeds per plant depending on the treatment) and germinated seeds (i.e., in which the radicle protruded from the seed coat) were counted. Then, four replicates of 25 seeds (gathered from unsprouted seeds collected from five plants per treatment) were sown in pleated filter paper, moistened with 40 mL deionized water, and placed in plastic boxes. The boxes were kept in incubators at 5°C and 20°C, respectively the sub- and optimum temperatures for oilseed rape germination (Schopfer and Plachy, 1984; ISTA, 2013). Observations were carried out two to five times per day depending on the temperature tested. After the germination tests, the germinated seeds were placed under natural light at 20°C for 7 days. Abnormal seedlings (seedlings with a radicle but without a hypocotyl, or with a thick glassy radicle and/or hypocotyl) were counted.

#### Conductivity measurements

For each treatment, measurements of electrolyte leakage were performed on 20 unsprouted individual seeds (*n* = 20). Seeds were previously weighed and individually soaked in 5 mL of deionized water at 20°C for 16 h. Conductivity was measured with a portable electrochemical analyzer (Consort C931, UK). Results are expressed in μS/cm/g of seed fresh weight.

### Statistical analyses

Results are expressed as the mean ± se of *n* replicates available per treatment. The normality of the distribution and equality of variances were previously tested (Kruskal-Wallis and Barlett tests respectively). Two-Way ANOVAs were performed for temperature (T), S, and T × S interaction effects on the measured variables (STATGRAPHICS Centurion XVI. II Software).

## Results

### Changes in yield components

The results of yield component analysis reflected the complex effects of T and S treatments on seed production. Globally, there was no significant difference in yield per plant (seed yield, Table 1) in the two T and S treatments and only a significant T × S interaction was observed (*P < 0.05*). Because seed yield is the result of two main components: the number of seeds per plant and SDW (for a single seed), yield variations should be analyzed along with the variations the number of seeds per plant and of SDW. The number of seeds per plant was significantly affected by both T and S treatments. It was the highest in Ctrl-HS (control seeds) and the lowest in HT-LS (double-stressed seeds). By contrast, SDW was less affected and had no significant effect in any treatment (Table 1). This was due to compensation effects between the two yield components: when the number of seeds decreased in HT-LS, the SDW was the highest (in trends, not significant). Variations in the number of seeds were correlated with variations in the number of reproductive branches (branches bearing pods >2 cm in length). High temperatures caused a significant decrease in the total number of branches and in the number of branches bearing mainly non-aborted pods (length >2 cm). No significant effects of S availability were observed on the number of branches, although a slight decrease in the total number of pods and a greater increase in non-aborted pods (>2 cm) were observed under S limitation without heat stress (Table 1). Heat stress and S restriction led to a significant increase in the number of 4–5-cm long pods on the main stem (Table 1), which can also be interpreted as compensation for a decrease in the total number of branches.

**Table 1:** **Mean values of yield components for the 4 modalities**.

	**Ctrl T-HS**		**HT-HS**		**Ctrl T-LS**		**HT-LS**		***T* effect**	***S* effect**	***T × S* effect**
	**mean**	***se***	**mean**	***se***	**mean**	***se***	**mean**	***se***			
Seed yield (mg plant^−1^)	4936	*627*	2940	*211*	3504	*526*	4027	*234*	2.6 ns	0.1 ns	7.8^*^
Number of seeds (plant^−1^)	2023	*85*	1508	*186*	1426	*56*	1399	*77*	5.4^*^	9.2^**^	4.4 ns
SDW (mg seed^−1^)	2.5	*0.4*	1.9	*0.1*	2.5	*0.2*	2.9	*0.2*	0.03 ns	4.0 ns	3.9 ns
**FINAL RAMIFICATION NUMBER**											
Total	8.7	*0.7*	5.0	*0.3*	7.5	*1.0*	5.6	*0.4*	18.1^***^	0.7 ns	0.2 ns
With pods > 2cm	7.5	*0.7*	4.0	*0.3*	5.5	*1.5*	3.8	*0.4*	11.7^**^	2.1 ns	1.4 ns
**POD NUMBER ON MAIN STEM PER CLASS LENGTH**											
<2cm (aborted)	2.3	*0.8*	2.4	*0.9*	5.5	*2.7*	0.6	*0.4*	3.7 ns	0.3 ns	3.9 ns
4–5 cm	1.0	*0.4*	2.4	*0.6*	2.0	*1.1*	5.2	*0.5*	13.8^**^	9.4^**^	2.1 ns

*Ctrl-T, Control Temperature; HT, High Temperature; HS, High Sulfur; LS, Low Sulfur*.

*SDW, seed dry weight; se, standard error (in italics)*.

F-values of the T, S and T × S effects are followed by the level of significance:

* P < 0.05,

** P < 0.01, and

*** P < 0.001;

*ns, non-significant*.

### Changes in grain composition

#### Different changes in seed C, N, and S contents under T and S treatments

While seed C and N contents remained stable whatever the treatment, S content was affected by S availability and to a lesser extent by high temperatures. S restricting conditions significantly reduced S content under both T treatments. Heat stress increased S content only under the LS treatment: in plants grown under HT-LS, the number of seeds per plant was reduced but the SDW and S content were higher (Tables 1, 2).

**Table 2:** **Mean values of measured variables related to grain composition and seed germination characteristics**.

	**Ctrl T-HS**		**HT-HS**		**Ctrl T-LS**		**HT-LS**		***T* effect**	***S* effect**	***T × S* effect**
	**mean**	***se***	**mean**	***se***	**mean**	***se***	**mean**	***se***			
**GRAIN COMPOSITION**											
**Elemental composition (%DW)**											
C content	54.4	*0.5*	53.1	*5.4*	54.0	*1.9*	54.6	*3.2*	0.1 ns	0.1 ns	0.3 ns
N content	4.9	*0.2*	4.7	*0.4*	4.7	*0.1*	5.0	*0.3*	0.01 ns	0.1 ns	3.5 ns
S content	0.52	*0.04*	0.53	*0.06*	0.36	*0.01*	0.45	*0.02*	7.9^*^	43.3^***^	3.9 ns
%Q^34^S	23.0	*2.9*	26.2	*0.6*	67.8	*2.1*	72.1	*1.2*	16.7^***^	2492^***^	0.4 ns
**Fatty Acids (FA) content (%DW)**											
Total FA	29.4	*3.5*	52.4	*6.4*	23.6	*2.2*	29.3	*3.6*	7.9^*^	5.6^*^	0.4 ns
C18:2/C18:3 (ω_6_/ω_3_)	2.47	*0.12*	1.82	*0.32*	3.11	*0.03*	1.70	*0.32*	17.2^***^	0.31 ns	0.15 ns
**Seed Storage Proteins (peak area)**											
Cruciferins (12S globulins)	0.35	*0.01*	0.29	*0.01*	0.43	*0.01*	0.36	*0.01*	48.9^***^	78.9^***^	0.9 ns
Napins (2S albumins)	0.19	*0.01*	0.21	*0.01*	0.16	*0.01*	0.16	*0.01*	4.17 ns	121.5^***^	4.2 ns
12S/2S ratio	1.79	*0.03*	1.40	*0.09*	2.77	*0.09*	2.32	*0.01*	31.2^***^	162.4^***^	0.4 ns
**Soluble sugars (%DW)**											
Sucrose	7.27	*0.210*	8.04	*0.172*	7.72	*0.394*	8.94	*0.164*	9.3^*^	4.2 ns	0.5 ns
Raffinose	0.206	*0.016*	0.201	*0.008*	0.156	*0.015*	0.187	*0.008*	0.7 ns	4.1 ns	1.2 ns
Stachyose	1.144	*0.062*	0.752	*0.047*	0.990	*0.061*	0.798	*0.034*	18.7^**^	0.6 ns	2.2 ns
(Raffinose + Stachyose)/ Sucrose	0.19	*0.01*	0.12	*0.01*	0.15	*0.01*	0.11	*0.01*	25.7^***^	0.1 ns	0.2 ns
**Hormone content (ng g^−1^ DW)**											
Abscissic acid (ABA)	87.6	*5.6*	17.9	*0.9*	69.8	*3.4*	35.4	*2.6*	191.0^***^	0.0 ns	21.9^*^
Gibberellic acid (GA3)	88.1	*3.5*	62.9	*3.1*	53.5	*3.5*	85.6	*7.4*	0.5 ns	1.6 ns	36.1^***^
ABA/ GA ratio	1.00	*0.07*	0.29	*0.02*	1.31	*0.05*	0.42	*0.03*	2291.0^***^	16.9^***^	0.1 ns
**SEED CHARACTERISTICS**											
Conductivity(μS cm^−1^ mg^−1^)	0.52	*0.11*	0.94	*0.12*	0.59	*0.14*	0.98	*0.13*	20.5^***^	0.3 ns	0.1 ns
Pre-harvest sprouting (%)	5.1	*2.1*	23.3	*7.6*	11.9	*3.5*	39.0	*11.1*	12.7^**^	3.2 ns	0.5 ns
% Abnormal seedlings	9	*4.1*	19	*4.7*	5	*2.5*	3	*1.0*	1.4 ns	8.6^*^	3.1 ns

Ctrl-T, Control Temperature; HT, High Temperature; HS, High Sulfur; LS, Low Sulfur. DW, dry weight; FA, fatty acid; SSP, Seed Storage Protein. se, standard error (in italics). F-values of the T, S and T × S effects are followed by the level of significance:

* P < 0.05,

** P < 0.01, and

*** P < 0.001;

*ns, non-significant*.

The %Q^34^S in seeds enabled estimation of the amount of ^34^S that was taken up during the labeling period (from the rosette stage GS16 to the beginning of pollination GS70) and translocated to the seeds at harvest (pod maturity, GS99). Under HS, most of the S in the seed came from S absorbed in the later stages after the beginning of pollination, while under LS, the S in the seed came from the remobilization of S, which was enhanced, as expected. Heat stress increased the %Q^34^S, showing that the S remobilization process was also enhanced by high temperatures (Table 2).

#### Marked changes in fatty acid (FA) and seed storage protein composition under both T and S treatments

#### Fatty acid composition

**Figure 1:**
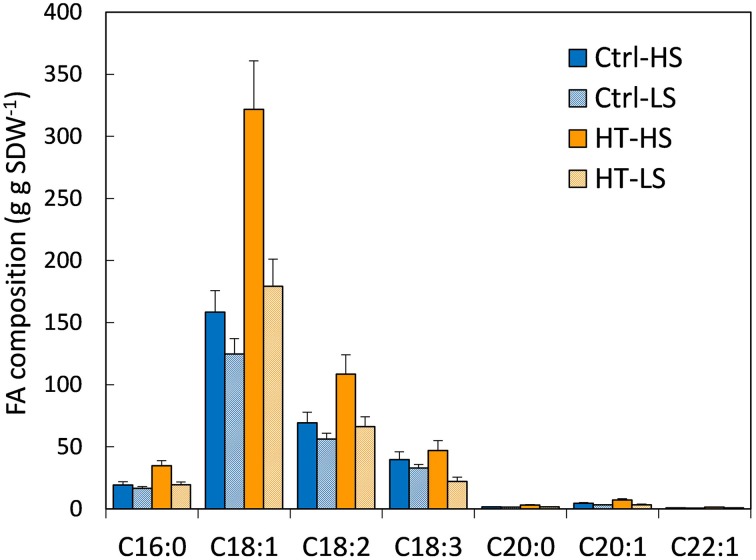
**FA composition (mg g SDW^−1^) under the 4 treatments**. Ctrl-T, Control temperature; HT, High temperature; HS, High sulfur; LS, Low sulfur. Bars denote s.e. C16:0 (palmitic acid), C18:1 (oleic acid), C18:2 (linoleic acid), C18:3 (linolenic acid), C20:0 (arachidic acid), C20:1 (gadoleic acid), C22:1 (erucic acid). Vertical bars are standard errors. Statistical effects are given in Supplemental Data Table 1.

Total FA contents were affected by both T and S treatments (Table 2). The highest FA content was measured under heat stress combined with non-limiting S conditions, and was almost twice the FA content in controls. A significant increase was also observed with LS under high temperatures, but to a lesser extent. A supply of S significantly increased total lipid contents. Unsaturated FAs (C18:1, C18:2, and C18:3 which belong to the ω_9_, ω_6_, and ω_3_ families, respectively) were the most abundant FAs, as expected, followed by the saturated FA, C16:0. The concentration of all FAs decreased under S stress whatever the T treatment (Figure 1). By contrast, the concentration of the main FAs, except C18:3, increased under high temperatures, whatever the S treatment. The C18:2/C18:3 (also denoted ω6/ ω3, Table 2) ratio is used as an indicator of nutritional quality of oil (the lower the ratio, the higher the nutritional value): it decreased significantly under heat stress under both S treatments, while no significant effect of S was observed (Table 2). Finally, the FA contents of seeds grown under the double-stress treatment (HT-LS) were closer to those in seeds grown under the non-stress treatment (Ctrl T-HS) than to those in seeds grown under single-stress treatments (Ctrl T-LS and HT-HS; Figure 1). Heat stress during pod filling partly mitigated the negative effects of S limiting conditions on the accumulation and composition of the FAs.

#### Seed storage proteins (SSPs)

While total N contents remained similar in all treatments, the amount of cruciferins (12S globulins) and napins (2S albumins) was impacted by heat stress and S restriction (Table 2). The cruciferin family contains fewer S-amino acids (methionine and cysteine) and thus fewer S-rich proteins than napins. The concentration of both cruciferins and napins were affected by S limitation, with higher concentrations of 12S globulins but lower concentrations of 2S albumins whatever the temperature treatment (Table 2). A significant effect of T was observed, but only on 12S globulins, with a decrease in their concentration under heat stress under both S supply treatments (Table 2). The 12S/2S ratio indicates protein quality in terms of S-containing essential amino acids by quantifying the relative amount of S-rich proteins i.e., the lower the ratio, the S-richer the protein. While S limitation increased the 12S/2S ratio, heat stress had the opposite effect by decreasing this ratio for a given S treatment.

### Changes in seed characteristics

#### Changes in soluble sugar contents and seed electrolyte leakage under heat stress indicated a potential decrease in seed storage capacity

While the C contents remained similar under the T and S treatments, changes in soluble sugar contents were observed with higher sucrose and lower stachyose contents under heat stress with both S supplies (Table 2). S limiting conditions emphasized the effects of temperature by increasing sucrose content. The (raffinose + stachyose)/sucrose ratio is correlated to seed storage ability in relation with seed desiccation conditions (a higher value is correlated with better storability). It decreased significantly in response to high temperatures regardless of the S supplies, and no significant S effects were observed (Table 2). Conductivity measurements also showed that high temperatures led to higher electrolyte leakage, which is consistent with the observed effects of HT on the (raffinose + stachyose)/sucrose ratio. Electrolyte leakage was about two times higher under HT (Table 2). No effect of S was observed although there was a slight increase in conductivity under S limitation for a given temperature treatment (Table 2).

#### High temperatures triggered accelerated seed germination, pre-harvest sprouting, and seedling abnormalities

**Figure 2:**
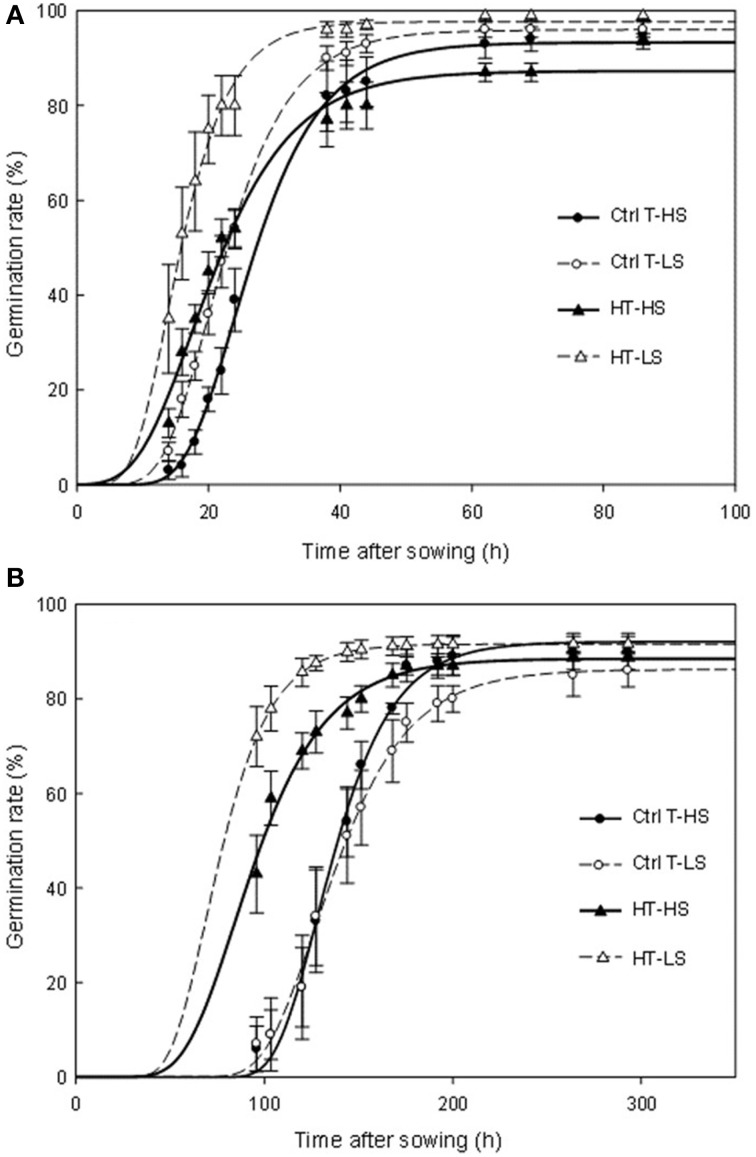
**Germination time courses at 20°C (A) and 5°C (B)**. Ctrl-T, Control temperature; HT, High temperature; HS, High sulfur; LS, Low sulfur. Vertical bars denote standard errors. Lines are fittings to a Gompertz function. *F*-values of the germination time courses and final germination percentages are given in Supplemental Data.

Pre-harvest sprouting (i.e., seeds that had germinated at harvest time) was observed in a small proportion in Ctrl T plants, indicating that the genotype Aviso was naturally prone to this phenomenon. Pre-harvest sprouting increased markedly under heat stress (Table 2). It was even higher under S restriction, although the S effect was not significant. The germination rates obtained for unsprouted harvested seeds at optimum (20°C) and sub-optimum (5°C) temperatures are presented in Figure 2. At 20°C, the final germination percentages were high (over 85%) with no significant differences between temperature treatments but significantly lower values under S restriction (Figure 2A, Supplemental Data Table 2). A significant T × S interaction effect was also observed. Heat stress and S restriction significantly accelerated germination time (Figure 2A) and the fastest germination rates were observed under the double-stress treatment (HT-LS). At 5°C, the final germination percentages were only slightly less than at 20°C and no differences were observed among treatments (Figure 2B, Supplemental Data Table 2). Germination took longer at 5°C than at 20°C, and seeds produced under heat stress germinated faster whatever the S treatment. Like for germination at 20°C, the fastest germination rates were observed under the double-stress treatment (HT-LS). Heat stress also caused higher percentages of abnormal seedlings, especially in the HT-HS treatment.

#### ABA content and ABA/GA ratio strongly decreased under high temperatures

ABA contents were considerably reduced under heat stress, with up to 4 times in the S-non limiting treatments, while no S effects were observed on ABA contents (Table 2). GA_3_ contents were not impacted by the S-limiting treatment or by heat stress, but a significant T × S interaction effect was observed because of the opposite effect of heat stress between the S treatments. Heat stress markedly reduced the ABA/GA ratio with both S supplies, while S limitation increased it to a lesser extent under both T treatments (Table 2). The decrease in the ABA/GA ratio under heat stress was thus lower in the S-limiting treatment than in the non-limiting S treatment (Table 2).

## Discussion

**Figure 3:**
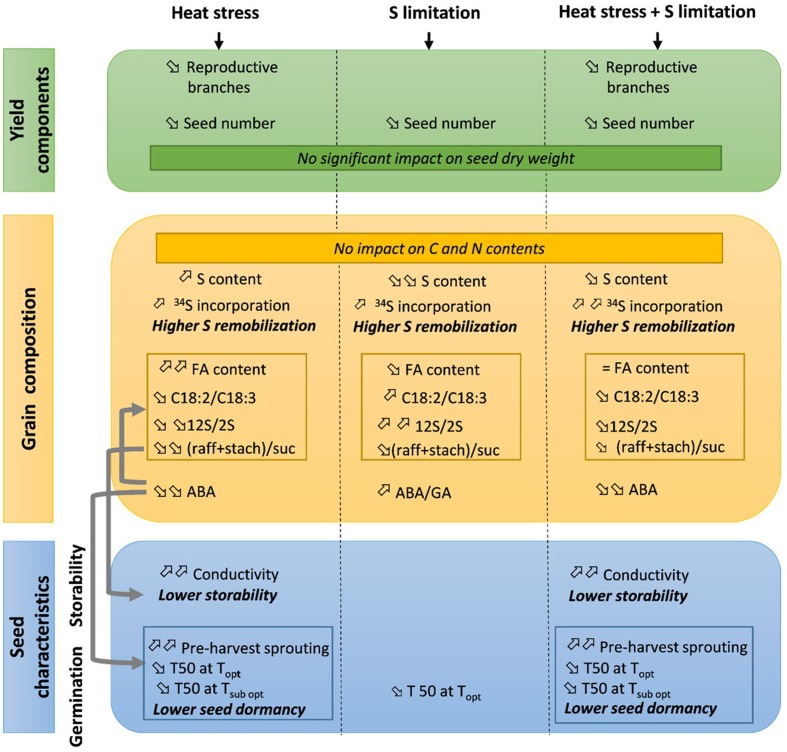
**Summary of the effects of heat stress and/or S limitation on yield components, seed composition, storability and germination in oilseed rape**. When a single effect (heat stress or S limitation) was observed, the trends (increase or decrease) are given in reference to the ANOVA in Tables 1, 2. When a double effect (heat stress + S limitation) is observed, the trends (increase or decrease) are given in reference to control plants (Ctrl T-HS). Gray arrows indicate known relations between variable levels. FA, fatty acids; raff, raffinose; stach, stachyose, suc, sucrose; ABA, absicic acid; GA, gibberelic acid; T50, time to reach 50% of germination; Topt, optimum temperature for germination (20°C); Tsubopt, sub-optimum temperature for germination (5°C).

The present study focused on the effects of two major environmental constraints during the grain filling period in oilseed rape: heat stress, which is expected to increasingly occur in the future, and reduction of S availability. In addition, these effects were analyzed on seed composition and nutritional value, as well as on seed storage ability and germination characteristics. Stress-induced modifications are usually analyzed separately, but in the context of climate changes, these effects need to be considered jointly. Our results suggest regulation by hormonal changes that might influence many seed characteristics simultaneously. The effects of heat stress, S restriction and their interactions are summarized in Figure 3.

### Both heat stress and sulfur restriction have a marked impact on FAs and SSPs and on the nutritional value of the seed

The effects of temperature can first be linked to the process of C fixation, assuming that the synthesis of FAs is determined by the production of carbohydrates and their allocation to the grain. Previous studies have shown that the final concentration of the grain oil is determined by the production of carbohydrates after flowering, and hence the amount of radiations intercepted during this period (Andrade and Ferreiro, 1996; Dosio et al., 2000; Izquierdo et al., 2008). In our study, high temperatures shortened the grain filling period and hence reduced the period of light interception. This impacted photosynthesis and the global production of carbohydrates, but not the C content of the individual seed. Our results indicated that C and N contents in seeds were not different among treatments. Only differences in FAs and SSPs were observed in seed contents among treatments. The processes of FA synthesis from carbohydrates were altered. The total FA amounts increased in response to elevated temperature for both S supplies, and decreased with a low S supply. In response to S limitation, the concentration of all the more abundant FAs decreased regardless of temperature. In previous studies on oilseed rape (Dubousset et al., 2010; D'Hooghe et al., 2014), early S restriction also reduced FA contents, mainly C18 derivatives. By contrast, C20:0 and C22:1 contents remained stable. This suggests that S restriction was unfavorable for FAs synthesis but not for chain elongation. Proteomics analyses of S-restricted seeds revealed that this modification in the FA balance was linked to disturbances in C metabolism and energy, and underlined the importance of acetyl-CoA, which is an S-containing metabolite required for the synthesis of FAs from pyruvate and for the chain elongation of FAs (D'Hooghe et al., 2014). Our results also showed that heat stress was rather beneficial because it allowed the C18:2/C18:3 ratio to be lowered, which is usually targeted to satisfy dietary requirements (Simopoulos, 2002). This effect of high temperatures is consistent with previous observations (Canvin, 1965). It should be noted that heat stress mitigated the negative effect of S restriction on the C18:2/C18:3 ratio, as double-stressed seeds (HT-LS) had lower C18:2/C18:3 ratios than single S-stressed seeds. Our results also revealed a lower relative proportion of C18:2 and C18:3 (as a percentage of total FAs, data not shown) under high temperatures, which is another targeted effect since reduced levels of polyunsaturated FAs are more suitable for frying oils (Matthaüs, 2006).

S restriction and also heat stress to a lesser extent, impacted seed S and SSP contents. Low S contents were associated with high levels of 12S/2S ratio. The decrease in the amount of napins (2S albumins) could be related to an impairment of Met and Cys synthesis due to a low S availability. The concomitant increase in cruciferin synthesis (12S globulins), which requires less S-containing amino acids than napins appeared to be a compensation and could explain why no differences were observed in total N content. These observations are consistent with previous results in oilseed rape, *Arabidopsis*, lupin, and pea (Higgins et al., 1986; Tabe and Droux, 2002; D'Hooghe et al., 2014), which showed that protein contents could be maintained under S restriction by decreasing the S-rich/S-poor protein ratio (D'Hooghe et al., 2014). By contrast, with S restriction, heat stress decreased the amounts of cruciferins but did not impact napins. Like the C18:2/C18:3 ratio, heat stress mitigated the negative effects of S restriction on the 12S/2S ratio as evidenced by the fact that double-stressed seeds (HT-LS) had lower 12S/2S ratios than single S-stressed seeds.

### Heat stress had major negative effects on seed storability and germination characteristics

Conductivity measurements showed increased electrolyte leakage from seeds produced under heat stress, indicating deterioration of cell membranes due to desiccation conditions during seed filling. Such increased seed leakage has been shown to be correlated with poor seed storage ability and with poor field emergence (Matthews and Powell, 2006; Demir et al., 2012; Mavi et al., 2014). These seeds could also be more prone to disease as their immediate environment during imbibition for germination is enriched in different ions that may favor the development of pathogens. The (raffinose + stachyose)/sucrose ratio is also an indicator of desiccation conditions during pod filling. Only heat stress had a negative impact with an increase in sucrose content along with a decrease in stachyose content leading to a decrease in the (raffinose + stachyose)/sucrose ratio. These results are consistent with previous observations in *Arabidopsis thaliana* (Kendall et al., 2011) and *Glycine max* (Keigley and Mullen, 1986), which also showed that high temperatures during seed development reduced seed quality and seed dormancy (for a review see Finkelstein et al., 2008).

Seed germination was also impacted by high temperatures and S restrictions during pod filling. High temperatures significantly increased pre-harvest sprouting, which means that high temperatures impaired the acquisition of seed dormancy. At the optimum temperature for germination (20°C) and even more at 5°C, seeds from heat stressed and S-limited plants germinated more rapidly. This reflects the lower level of dormancy of seeds produced under heat stress. Under high temperatures, very high rates of abnormal seedlings were also observed. These results should be interpreted jointly as detrimental effects of heat stress caused high pre-harvest sprouting rates and, in the remaining unsprouted seeds, abnormal seedling rates reached 20% under the non-limiting S treatment. Abnormal seedlings rates were also increased by S restriction, but to a lesser extent. Previous works showed a correlation between seed S content and time to germinate: the lowest the S content, the longer the time for germination (Eggert and von Wirén, 2013). D'Hooghe et al. (2014) also reported longer germination time for seeds produced under S limiting conditions. However, in our study, we observed lower germination times for seeds produced under LS conditions. These seeds has also similar soluble sugars contents to seeds produced under non-limiting S conditions, which might indicate that their level of dormancy was not impacted. This was consistent with studies on *Medicago truncatula* that showed a correlation between longer time for germination and low seed contents of sucrose and sugars of the raffinose family oligosaccharides (RFO) because they can be quickly degraded for expansion and growth requirements (Zuber et al., 2013). Therefore, we can assume that in our conditions, S limitation had triggered hormonal changes which counterbalanced the expected effects on seed dormancy and germination in seeds with low S contents.

### Hormonal disturbances may explain the modifications in germination characteristics and grain composition

Our results showed that the ABA/GA ratio decreased under heat stress, which is consistent with higher pre-harvest sprouting scores, increased germination speed, and high rates of seedling abnormalities. Increases in the ABA/GA ratio have been shown to be involved in germination inhibition and maturation induction (Koornneef et al., 1982; White et al., 2000). ABA content was correlated with variations in dormancy in seeds with a similar degree of ABA sensitivity (Hilhorst, 1995; De Castro and Hilhorst, 2000). These observations were also supported by gene expression studies during seed maturation in *Brassica napus* L. They highlighted the role of ABA related genes in the induction of secondary dormancy known to be induced after exposure to stressful environmental conditions in rapeseed (Pekrum et al., 1997; Momoh et al., 2002; Gulden et al., 2004a,b; Fei et al., 2007).

The level of ABA was also showed to influence the deposition of seed compounds during grain filling i.e., FAs (Chandrasekaran et al., 2014), SSPs (Crouch and Sussex, 1981; Finkelstein et al., 1985) and soluble sugars (Finkelstein and Gibson, 2001; Rolland et al., 2002; Dekkers and Smeekens, 2007). Studies that focused on gene expression profiling in developing *Brassica napus* L. seeds highlighted differential expressions of genes related to seed storage proteins, carbohydrate, and lipids metabolisms according to the seed developmental stage (Yu et al., 2010). Therefore, the timing of stressful conditions throughout the pod filling period might be determining since it could impact the biosynthesis of specific storage compounds. Yu et al. (2010) also reported a number of regulatory and signaling genes related to seed development and storage compounds, amongst them transcription factors (TFs) such as *ABI5* which is involved in ABA signaling. Moreover, the role of TFs, as master regulators of gene expression, in crosstalk with abiotic stress including heat was largely described (for review, Nakashima et al., 2014). This evidences that changes in ABA content for seeds produced under HT conditions might modified the downstream expression of genes associated to the synthesis of seed storage compounds.

In our study, ABA contents were only measured in the dry seeds at the end of grain filling, but differences in treatments were fortunately still visible. It would be interesting to measure the dynamics of the hormonal changes during seed filling and to test whether genotypic differences exist in response to heat stress in both seed characteristics (composition of seed storage compounds and germination).

In conclusion, changes were observed not only in the concentrations but also in the ratios of nutritional compounds (FAs, SSPs, and soluble sugars), which are indicators of grain quality and seed storage capacity. Overall, heat stress decreased yield, but improved important ratios that determine the quality of the oil and meal (lipids and proteins) and also digestibility for cattle (soluble sugars) by contrast to S limitation. Under heat stress, concentrations of the first synthesized compounds (such as saturated FAs and C18:1 for FAs, and sucrose for soluble sugars) increased while the more complex compounds obtained by desaturation and/or elongation of the first synthesized ones (such as C18 derivatives for FAs, and raffinose and stachyose for soluble sugars) decreased.

Adequate control of S fertilization under heat stress could help improve grain quality and limit the detrimental effects of climate changes. Previous studies on the SSPs in a panel of cultivars highlighted marked variations in cruciferins and napins contents, ranging from 32 to 53% and 25 to 45% of the total SSPs, respectively (Malabat et al., 2003). This suggests that natural variations may also occur in response to heat stress with regards to seed composition and should be studied. Moreover, monitoring the dynamics of ABA contents during seed filling would help to interpret modifications in grain composition and in germination characteristics, since changes in ABA levels appear to control pathways related to the accumulation of lipids, SSPs and soluble sugars, as well as seed germination and seed storage ability. Different durations and intensities of heat stress should also be tested along with distinct crop stages for S limitation, as the effects of S deficiency on yield and grain quality are tightly linked to the physiological stages in oilseed rape (Dubousset et al., 2010; D'Hooghe et al., 2014; Girondé et al., 2014). Finally this work revealed contrasting effects of heat stress and S restriction on grain quality and germination characteristics. They should thus be considered jointly when monitoring S fertilization in the context of climate change.

## Author contributions

SB, PD, JT, JA and PE contributed to the experimental design, to plant growth and tissue sampling and have been involved in revising the article for important intellectual content. CD supervised the choice of relevant measurements on seeds. MB made the spectrometry analysis. CL was involved in the SSP analyses, interpretation of protein data and revising the manuscript. TWK was involved in the hormone measurements. SM performed the whole raw data analysis (including statistical analyses). SM and CD made interpretation of data and writing of the article.

### Conflict of interest statement

The authors declare that the research was conducted in the absence of any commercial or financial relationships that could be construed as a potential conflict of interest.
