# Corrigendum: Heat Stress during Seed Filling Interferes with Sulfur Restriction on Grain Composition and Seed Germination in Oilseed Rape (*Brassica napus* L.)

**DOI:** 10.3389/fpls.2015.01236

**Published:** 2016-01-12

**Authors:** Sophie Brunel-Muguet, Philippe D'Hooghe, Marie-Paule Bataillé, Colette Larré, Tae-Hwan Kim, Jacques Trouverie, Jean-Christophe Avice, Philippe Etienne, Carolyne Dürr

**Affiliations:** ^1^UCBN, INRA, UMR INRA–UCBN 950 Ecophysiologie Végétale, Agronomie and Nutritions N.C.S., Normandie UniversityCaen, France; ^2^INRA UR1268 BIA, Rue de la GéraudièreNantes, France; ^3^Department of Animal Science, Environment-Friendly Agriculture Research Center, Institute of Agricultural Science and Technology, College of Agriculture and Life Science, Chonnam National UniversityGwangju, South Korea; ^4^INRA, UMR 1345, Institute of Research on Horticulture and SeedsBeaucouzé, France

**Keywords:** oilseed rape, sulfur, temperature, germination, grain quality, lipids, proteins, sugars

Reason for Corrigendum:

After our article was published online, it was brought to our attention that the values of the ratio ω6/ω3 in **Table 2** were inverted between the four treatments i.e., Ctrl T-HS, HT-HS, Ctrl T-LS, and HT-LS. All of the other data related to the total FAs and the *F*-values for the FAs (in the Supplemental data, **Table 2**) are correct.

Corrections for the values of the ratio ω6:ω3 follow:

**Table:** 

	**Ctrl T-HS**		**HT-HS**		**Ctrl T-LS**		**HT-LS**		**T effect**	**S effect**	**T × S effect**
	**mean**	**se**	**mean**	**se**	**mean**	**se**	**mean**	**se**			
C18:2/C18/3 (ω6/ω3)	1.82	0.32	2.47	0.12	1.70	0.03	3.11	0.32	17.2^***^	1.09 ns	2.24 ns

Therefore, high temperature increased the ratio for both S supplies condition.

The authors apologize for the mistake that changes the conclusion with regards to the effect of temperature on this ratio.

In the text and in Figure 3, we want to make the following modifications:

**Figure 3:**
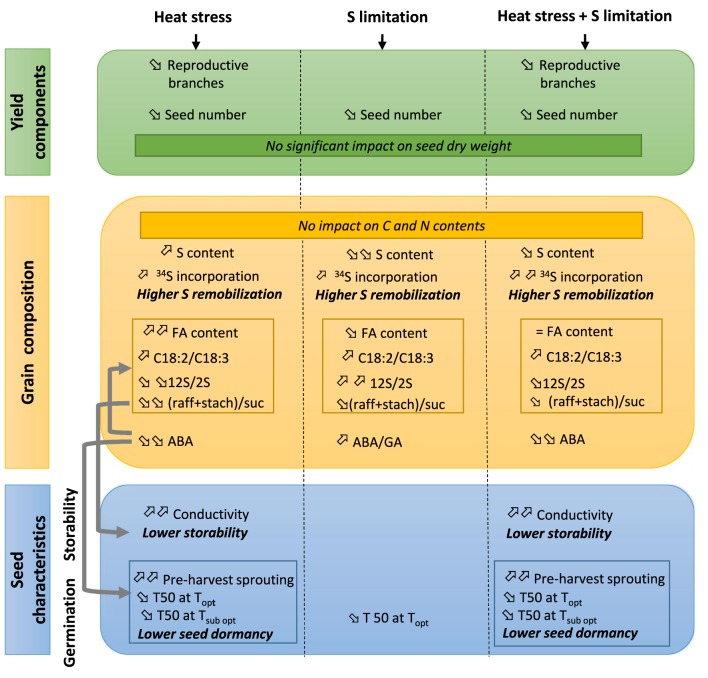
**Summary of the effects of heat stress and/or S limitation on yield components, seed composition, storability, and germination in oilseed rape**. When a single effect (heat stress or S limitation) was observed, the trends (increase or decrease) are given in reference to the ANOVA in **Tables 1, 2**. When a double effect (heat stress + S limitation) is observed, the trends (increase or decrease) are given in reference to control plants (Ctrl T-HS). Grey arrows indicate known relations between variable levels. FA: fatty acids; raff: raffinose; stach: stachyose, suc: sucrose; ABA: absicic acid; GA: gibberelic acid; T50: time to reach 50% of germination; Topt: optimum temperature for germination (20°C); Tsubopt: sub-optimum temperature for germination (5°C)

In the abstract:

Instead of “While LS had negative effects on seed composition by reducing the FA contents and increasing the ratio S-poor SSPs (12S globulins)/S-rich SSPs (2S albumins) ratio, HT had positive effects by increasing S and FA contents and decreasing the C18:2/C18:3 ratio and the 12S/2S protein ratio,” we want to write: “While LS had negative effects on seed composition by reducing the FA contents and increasing the ratio S-poor SSPs (12S globulins)/S-rich SSPs (2S albumins) ratio, HT had positive effects by increasing S and FA contents and decreasing the 12S/2S protein **but it negatively affected the C18:2/C18:3 ratio by increasing it.”**

In the result section:

Page 5 line 500: Instead of “The C18:2/C18:3 (also denoted ω6/ ω3, **Table 2**) ratio, which is used as an indicator of nutritional quality of oil (the lower the ratio, the higher the nutritional value), decreased significantly under heat stress under both S treatments, while no significant effect of S was observed (**Table 2**),” we want to write “The C18:2/C18:3 (also denoted ω6/ ω3, **Table 2**) ratio, which is used as an indicator of nutritional quality of oil (the lower the ratio, the higher the nutritional value), **increased** significantly under heat stress under both S treatments, while no significant effect of S was observed (**Table 2**).”

In the discussion section:

Page 8 lines 846–849: Instead of “Our results also showed that heat stress was rather beneficial because it allowed the C18:2/C18:3 ratio to be lowered, which is usually targeted to satisfy dietary requirements (Simopoulos, 2002),” we want to write “Our results also showed that heat stress **was rather negative because it increased the C18:2/C18:3 ratio**, **which is usually not targeted with regards to dietary requirements** (Simopoulos, 2002).” Therefore, the following sentence is wrong “it should be noted that heat stress mitigated the negative effect of S restriction on the C18:2/C18:3 ratio, as double stress (HT-LS) had lower C18:2/C18:3 ratios than single stressed seed.

Page 9 line 920: Instead of “Like the C18:2/C18:3 ratio, heat stress mitigated the negative effects of S restriction (…),” we want to write “Heat stress mitigated the negative effects of S restriction….”

Page 10 line 1030: instead of “Overall, heat stress decreased yield, but improved important ratios that determine the quality of the oil and meal (lipids and proteins),” we want to write: “Overall, heat stress decreased yield, but improved an important ratio that determine the quality meal (proteins).”

In Figure 3

The changes deal with the arrows for the C18:2/C18:3 ratios that increased with Heat Stress (first column) and with Heat Stress+ S limitation (third column).

In addition to these points above, the authors want to correct the followings sentences:

- Page 2 line 148 (at the end of the first paragraph of the introduction): “S limitation may lead to a loss of 40% of seed yield in oilseed rape” instead of “S limitation may lead to 40% seed yield in oilseed rape.”

## Author contributions

SB, PD, JT, JA, and PE contributed to the experimental design, to plant growth and tissue sampling and have been involved in revising the article for important intellectual content. CD supervised the choice of relevant measurements on seeds. MB made the spectrometry analysis. CA was involved in the SSP analyses, interpretation of protein data, and revising the manuscript. TK was involved in the hormone measurements. SB performed the whole raw data analysis (including statistical analyses). SB and CD made interpretation of data and writing of the article.

### Conflict of interest statement

The authors declare that the research was conducted in the absence of any commercial or financial relationships that could be construed as a potential conflict of interest.

