# Supplementary material for: Heat stress during seed filling interferes with sulfur restriction on grain composition and seed germination in oilseed rape (Brassica napus L.)
Source: Front Plant Sci. 2015 Apr 10;6:213. doi: 10.3389/fpls.2015.00213 (PMC4392296; doi:10.3389/fpls.2015.00213)
Supplement: Supplementary file 1 [file Table1.DOCX]

**Supplemental data 1:**

| Treatment effect | | T | | S | | T x S | |
| --- | --- | --- | --- | --- | --- | --- | --- |
| ***Grain quality:*** Fatty acids (FA) | |  | |  | |  | |
| C16 palmitic acid | | 9.8** | | 9.6** | | 4.6* | |
| C18:1 oleic acid | | 17.2*** | | 11.3** | | 4.3 ns | |
| C18:2 linoleic acid | | 5.4* | | 6.7* | | 1.9 ns | |
| C18:3 α-linolenic acid | | 0.1 ns | | 7.3* | | 2.4 ns | |
| C20 arachidic acid | | 7.6* | | 10.0** | | 4.0 ns | |
| C20:1 gadoleic acid | | 3.5 ns | | 12.3* | | 3.1 ns | |
| C22:1 erucic acid | | 2.1 ns | | 6.8* | | 1.9 ns | |
| C18:2/C18:3 | | 17.2*** | | 0.31 ns | | 0.15 ns | |

F-values of temperature (T), effects of sulfur (S) and of T x S on FA composition and proportions of total FA for C18:1, C18:2, C18:3 and on ratio of C18:2/C18:3
