# Supplementary material for: Heat stress during seed filling interferes with sulfur restriction on grain composition and seed germination in oilseed rape (Brassica napus L.)
Source: Front Plant Sci. 2015 Apr 10;6:213. doi: 10.3389/fpls.2015.00213 (PMC4392296; doi:10.3389/fpls.2015.00213)
Supplement: Supplementary file 2 [file Table2.DOCX]

**Supplemental data 2:**

Mean (± se) and F-values of temperature (T), effects of sulfur (S) and of T x S on T50 and final rate of germination at 20 °C and 5 °C.

| Seed germination characteristics | Ctrl T-HS | | HT-HS | | Ctrl T-LS | | HT-LS | | T | S | T x S |
| --- | --- | --- | --- | --- | --- | --- | --- | --- | --- | --- | --- |
|  | mean | se | mean | se | mean | se | mean | se |  |  |  |
| ***Seed germination*** |  |  |  |  |  |  |  |  |  |  |  |
| Germination at 20 °C |  |  |  |  |  |  |  |  |  |  |  |
| T50 | 27.6 | 0.3 | 22.1 | 0.1 | 22.9 | 0.1 | 15.8 | 0.2 | 19.1*** | 14.2** | 0.3 ns |
| Final rate | 94.0 | 0.3 | 86.3 | 0.3 | 96.0 | 0.2 | 97.7 | 0.1 | 2.1 ns | 10.8** | 5.3* |
| Germination at 5 °C |  |  |  |  |  |  |  |  |  |  |  |
| T50 | 137.1 | 0.8 | 98.9 | 0.6 | 142.4 | 1.1 | 78.1 | 0.8 | 53.5*** | 1.3 ns | 3.7 ns |
| Final rate | 90.9 | 0.5 | 88.7 | 0.3 | 85.8 | 0.5 | 92.7 | 0.2 | 0.5 ns | 0.0 ns | 1.9 ns |
